# Pacing for Vasovagal Syncope

**Published:** 2002-10-01

**Authors:** Nevin T Wijesekera, Arvinder S Kurbaan

**Affiliations:** 1 Department of Medicine, Kingston Hospital, Surrey, KT2 7QB, UK; 2 Department of Cardiology, London Chest Hospital, London, E2 9JX, UK

## Introduction

Vasovagal syncope is a common condition, usually associated with a benign prognosis. Most sufferers experience only occasional symptoms, and can be treated with reassurance and lifestyle advice. However, a minority of patients are debilitated by frequent fainting that can infringe on daily living, or even mimic sudden death. This has been termed "malignant" vasovagal syncope because of the associated falls and physical injury. In these cases, a more interventional approach may be appropriate.

Pharmacological measures have been the mainstay of treatment for recurrent vasovagal syncope: beta-blockers (e.g. atenolol), serotonin reuptake inhibitors (e.g. paroxetine), certain vasoconstricting drugs (e.g. midodrine) and fluid retaining agents (e.g. fludrocortisone) have been of particular interest. However, there is only mixed support from randomised controlled trials for the efficacy of these agents in preventing vasovagal syncope [1-3].

In the last few years, cardiac pacing has been advocated for the treatment of some forms of vasovagal syncope. This article reviews the literature and discusses the indications for pacing in vasovagal syncope.

## Rationale for pacing

Vasovagal syncope results from transient dysfunction of autonomic cardiovascular regulation. Haemodynamic collapse, resulting in either syncope or presyncope, may be induced on tilt table testing. The most readily quantifiable physiological responses are vasodepression (arterial blood pressure fall) and cardioinhibition (heart rate fall). Cardiac pacing aims to overcome bradycardia during syncope and provide enough heart rate support to compensate for the hypotension.

## Evidence for pacing

### Temporary pacing studies

Since the early 1990s, pacing has been an accepted treatment for selected patients with vasovagal syncope by both the British Pacing Electrophysiology Group and American Heart Association/American College of Cardiology guidelines [4,5]. These recommendations were based on the results of several non-randomised observational studies. These studies generally indicated a beneficial role for temporary pacing during tilt table testing  [6,7] Some investigators, such as Sra et al. [8], reported results that could be interpreted as negative because pacing failed to consistently abort syncope, although many patients (18 of 22) who initially had syncope had only presyncope on repeat testing.

### Non-randomised studies with permanent pacing

Following these initial investigations, evidence to support the use of permanent pacemakers in vasovagal syncope came with the publication of three studies which used historical controls (Table 1) [9-12]. Dual-chamber pacemakers were implanted in a total of 77 patients, the majority of whom had demonstrated bradycardia on tilt table induced syncope. These studies consistently showed that after insertion of a permanent pacemaker, most patients either no longer had syncope or had far fewer episodes of syncope.

### Randomised studies with permanent pacing

More recently support for a beneficial role for pacing has come from three randomised controlled studies (Table 2). Patients were selected if they had a positive tilt table test with a predefined severity of bradycardia. The first Vasovagal Pacemaker Study (VPS 1) [13] included a more highly symptomatic population than either the Vasovagal Syncope International Study (VASIS) [14] or the Syncope Diagnosis and Treatment Study [15]: 6 attacks per year versus 3 attacks in 2 years. In VPS 1, patients were randomised either to receive a pacemaker with automatic rate drop responsiveness or to receive optimal medical therapy as determined by the treating physician. The study was designed to enroll 248 patients but was stopped when the interim analysis of 54 patients fulfilled the predefined criteria for early termination on the grounds of efficacy. There was a significant reduction in the time to first recurrence of syncope in those allocated to pacing compared with medical therapy (22% vs 70%; P=0.0002).

VASIS compared dual-chamber pacing with rate hysteresis with no pacemaker implantation. During a mean follow-up period of 3.7±2.2 years, there was a lower rate of recurrent syncope in the pacemaker arm than in the no-pacemaker arm (5% vs 61%; P=0.0006).

The Syncope Diagnosis and Treatment Study assessed whether dual-chamber pacing with rate drop response or atenolol best prevented vasovagal syncope. All patients were older than 35 years, had >3 syncopal episodes in the preceding 2 years, and had a positive tilt table test with a bradycardia less than 60 bpm. The study was stopped after the first interim analysis of efficacy because those randomised to a pacemaker fainted less frequently than those randomised to atenolol (4% vs 26%; P=0.004).

In summary, these three studies showed that, in carefully selected patients, pacing is beneficial. However, as these were open-label studies, a placebo effect of pacemaker implantation cannot be excluded. The ongoing second Vasovagal Pacemaker Study (VPS II) is a multinational randomised clinical trial that will enroll 100 patients over three years and will address whether dual-chamber pacing with rate drop sensing is superior to placebo. Patients are eligible if they have had six or more syncopal episodes and a positive tilt table test, although development of bradycardia is not an essential requirement for inclusion. All patients receive a dual-chamber pacemaker with rate drop capabilities; half are randomised to rate drop sensing, the other half are randomised to a mode which senses and records heart rate periods but does not pace. The primary outcome is the first recurrence of syncope. Patients may then enter a second phase in which they are randomised to pacing with or without rate drop sensing. Enrolment closed in October 2001, and results are anticipated to be available by late 2002.

## The optimal mode for sensing vasovagal syncope onset

Early detection of impending vasovagal syncope is a key factor in the development of an effective pacing strategy. Unlike other conditions requiring pacing, the fall in heart rate during vasovagal syncope is often insidious rather than abrupt. Pacemakers with a rate drop response algorithm are therefore considered particularly appropriate as they take account of the rate of fall, as opposed to the more conventional rate hysteresis systems that pace when a particular heart rate is reached. In a randomised trial, Ammirati et al. compared rate drop responsiveness and rate hysteresis in 20 patients with recurrent syncope [16]. This study demonstrated a benefit for those with rate drop responsiveness (0/12 fainted) compared with rate hysteresis (3/8 fainted). The second phase of VPS II hopes to establish whether dual-chamber pacing with rate drop sensing is superior to dual-chamber pacing at an escape rate of 50 beats per minute.

Further refinement in the ability to detect incipient syncope may arise from the recognition of other sensing strategies, such as changes in the QT interval, right ventricular pressure, central venous temperature or changes in respiratory pattern. Minute volume sensing together with heart rate change may offer earlier detection of impending vasovagal syncope than can heart rate alone [17]. Pacemakers with this facility are now available, and could prove to be a significant adjunct to current sensing modes.

## The optimal mode for pacing in vasovagal syncope

Early studies showed single-chamber ventricular demand pacing (VVI) to be ineffective in preventing vasovagal syncope [7,18]. The absence of atrioventricular synchrony appears to aggravate the peripheral vasodilatation, perhaps by retrograde activation of atria and release of natruretic peptides. Invasive haemodynamic studies have demonstrated that dual-chamber pacing achieves a reduction in the rate of fall of arterial pressure as heart rate drops, which in the clinical setting may sufficiently prolong consciousness to allow injury to be avoided [6,7]. McLeod et al. assessed the relative usefulness of single-chamber pacing (VVI) and dual-chamber pacing (DDD) in the prevention of vasovagal syncope in 12 highly symptomatic young children [19]. In a three way, double blind randomised crossover design, the pacemakers were programmed to no pacing, ventricular pacing with rate hysteresis, or dual-chamber pacing with rate drop responsiveness. Each treatment exposure lasted 4 months. Both pacing modes were equivalent, and more effective than no pacing, in preventing syncope. DDD pacing was superior to VVI pacing in preventing presyncope. Dual-chamber pacing has now been clinically assessed in randomised trials of pacing in vasovagal syncope, and is generally considered to be the pacing mode of choice (Table 3) [13-15]. However, the optimal pacemaker intervention rate is still the subject of debate. It has been suggested that high rate intervention (≥120 beats/min ) may be better than standard rate pacing (80-90 beats/min) in improving symptoms and/or aborting syncope [20].

## Selecting patients with vasovagal syncope for pacing

The crucial issue is to identify individuals who could benefit from pacing. Pacemakers should be considered for patients with frequent and medically refractory vasovagal syncope in whom there is evidence for bradycardia. Patients with specific drug intolerances or contraindications may be considered earlier. The VASIS group proposed a classification of the haemodynamic collapse patterns seen on tilt testing for the purpose of identifying potential candidates for drug or pacemakers trials (Table 4) [21]. Patients with predominant bradycardia (cardioinhibition) are the target for pacing. Within this group there is further subdivision into more severe or less severe forms. The expectation is that those with the more severe form will derive the greatest benefit from pacing. However, there is concern with pacing this group as it appears that more severe cardioinhibition is more prevalent in the younger population. Pacing young people has a considerable long-term burden, not least of which is the need for periodic system replacement. The other group that may benefit from pacing are those with chronotropic incompetence. This usually affects a much older population, so there is less reluctance to pace these patients. More recently, data from the International Study on Syncope of Uncertain Etiology suggests that the spontaneous syncopal event may be the result of a more severe bradycardia than that reproduced during tilt testing [22]. Hence, more patients may benefit from pacing than predicted by tilt testing. These findings need to be supported by further studies.

## Combination therapy

Cardiac pacing cannot address the profound vasodilatation that occurs together with cardioinhibition in vasovagal syncope. Therefore, pacing should not be seen in isolation or always as an alternative to pharmacological intervention. Recently, there has been interest in a combined approach, using pharmacological support for the vasodepressor component and pacing for the cardioinhibitory component - for example, fludrocortisone to minimise intravascular volume depletion and pacing to modify the heart rate response. Novel therapies, such as local drug delivery systems, may alter the role of pacing or even supersede pacing for vasovagal syncope. In a small study, Giada et al. assessed an implantable device that delivers intravenous phenylephrine when activated at the onset of syncope (prodromal symptoms with hypotension) [23]. Tilt-induced syncope was aborted in 94% of treated patients, but not at all when a placebo was delivered. In the future, with greater understanding of the underlying pathophysiology, the appropriate treatment may be tailored to the individual patient.

## Conclusion

Patients with frequent fainting have a poor quality of life. There is increasing evidence that pacing can prevent or delay fainting in selected patients with medically refractory vasovagal syncope. Those with absolute or relative bradycardia on tilt table testing are most likely to benefit. Dual-chamber pacing with a rate drop response algorithm appears to be the optimal protocol. On-going trials will clarify unresolved issues of how and when to pace.

## Figures and Tables

**Table 1:**
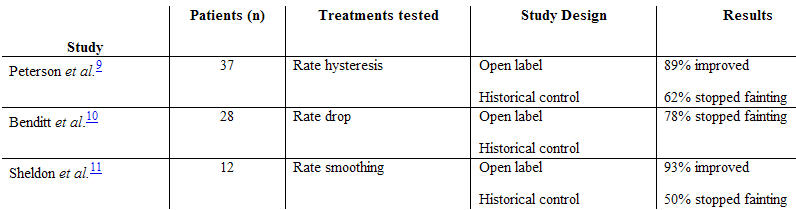
Non-randomised studies with permanent pacing

**Table 2:**
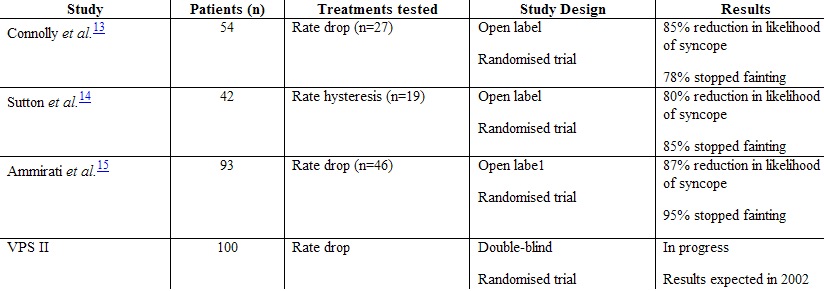
Randomised trials with permanent pacing

**Table 3:**
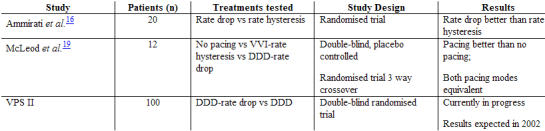
Studies comparing sensing and pacing modes

**Table 4:**
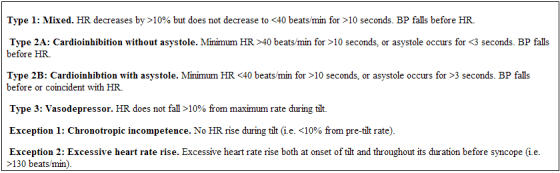
VASIS classification of positive responses to tilt testing
